# Rapid direct disk diffusion testing for antibiotic resistance in urinary tract infections: a bacterial concentration-adjusted approach

**DOI:** 10.1128/spectrum.00888-25

**Published:** 2025-09-22

**Authors:** Henning Sabersky-Müssigbrodt, Seoras Russell, Nina Wantia, Oliver Hayden

**Affiliations:** 1 Heinz Nixdorf Chair of Biomedical Electronics, School of Computation, Information and Technology, TranslaTUM, Technical University of Munich 663294, Munich, Germany; 2 Institute for Medical Microbiology, Immunology and Hygiene, Technical University of Munich 197674, Munich, Germany; 3 Munich Institute of Biomedical Engineering, Technical University of Munich 675423, Munich, Germany; UCI Health, Orange, California, USA

**Keywords:** urinary tract infection, bacteriuria, antibiotic susceptibility testing, direct susceptibility testing, disk diffusion test, 0.5 McFarland, inoculum effect

## Abstract

Urinary tract infections are among the most prevalent bacterial infections worldwide, typically diagnosed using clinical symptoms, dipstick tests, and laboratory methods requiring standardized bacterial suspensions for antibiotic susceptibility testing (AST). This study evaluated two rapid disk diffusion methods for urine samples that incorporate bacterial concentration into the analysis, eliminating the need for standardized suspensions. The threshold-adapted approach compares inhibition zones to concentration-specific breakpoints derived from reference strains, while the regression-based method transforms inhibition zones at various concentrations into predicted standard (0.5 McFarland) values using a linear model. In both methods, urine and antibiotic disks are applied to one agar plate for disk diffusion, while a separate plate determines bacterial concentration, enables isolation, and supports matrix-assisted laser desorption/ionization-time-of-flight (MALDI-TOF) analysis. This simultaneous processing reduces diagnostic time by 18–24 hours. The approaches were compared with traditional disk diffusion testing using defined bacterial suspensions, reference strains, and clinical urine samples. In trials using defined suspensions from 0.5 McFarland to 10**^3^** CFU/mL, the threshold-adapted method achieved 94.7% and 94.1% categorical agreement for susceptible and resistant bacteria, respectively; the regression-based method achieved 100% for susceptible and 88.2% for resistant bacteria. When urine was applied directly, both methods showed 93.7% true susceptible and 94.1% true resistant agreement with standard testing. By integrating inhibition zone size, antibiotic, species, and bacterial concentration, these rapid AST methods streamline urine diagnostics and show high agreement with standard testing—highlighting their potential as practical alternatives to conventional AST, particularly in resource-limited settings where reduced diagnostic time and simplified laboratory procedures can significantly improve patient care.

**IMPORTANCE:**

Antibiotic susceptibility testing (AST) for urinary tract infections typically requires time-consuming standardization of bacterial suspensions, delaying targeted treatment. Prior direct susceptibility testing (DST) approaches have largely overlooked the inoculum effect, testing at a single, arbitrary concentration and risking misclassification of both resistant and susceptible isolates. This study presents two rapid disk diffusion methods that systemically incorporate bacterial concentration into the analysis, enabling direct testing from urine samples without prior inoculum adjustment. Both approaches demonstrated high agreement with standard AST and reduced diagnostic time by up to 24 hours. These concentration-aware methods may streamline susceptibility testing workflows, particularly in resource-limited settings, and represent a practical advancement toward faster, clinically reliable DST.

## INTRODUCTION

Urinary tract infections (UTIs) are among the most prevalent infectious diseases, with an estimated 400 million cases annually (1). The diagnosis of uncomplicated UTIs is often constrained by the resources required for urine cultures, leading to a reliance on patient symptoms and urine dipstick tests. Urine cultures, which can identify pathogens and potential resistance, are typically reserved for cases of unsuccessful treatment, high-risk groups, or complicated UTIs (2). This diagnostic approach, recommended by guidelines but not tailored to individual patients, heightens the risk of incorrect antibiotic treatment. This, in turn, increases the potential for complications and the development of antibiotic resistance (3–7).

While modern laboratories primarily determine the minimum inhibitory concentration (MIC) using automated systems, the disk diffusion test remains a crucial and cost-effective component of antibiotic susceptibility testing (AST), particularly in resource-poor regions (8, 9). The disk diffusion method involves inoculating an agar plate with a standardized bacterial suspension, which requires prior overnight incubation of the urine sample and isolation of the bacteria before placing an antibiotic disk on the plate. The antibiotic diffuses into the culture medium, and after 18–24 hours of incubation, the inhibition zone diameter is measured. This value is compared with breakpoint tables published by institutions such as Clinical and Laboratory Standards Institute (CLSI) or European Committee on Antimicrobial Susceptibility Testing (EUCAST). These tables provide threshold values for different bacterial genera and antibiotics to indicate resistance (10, 11).

**Fig 1:**
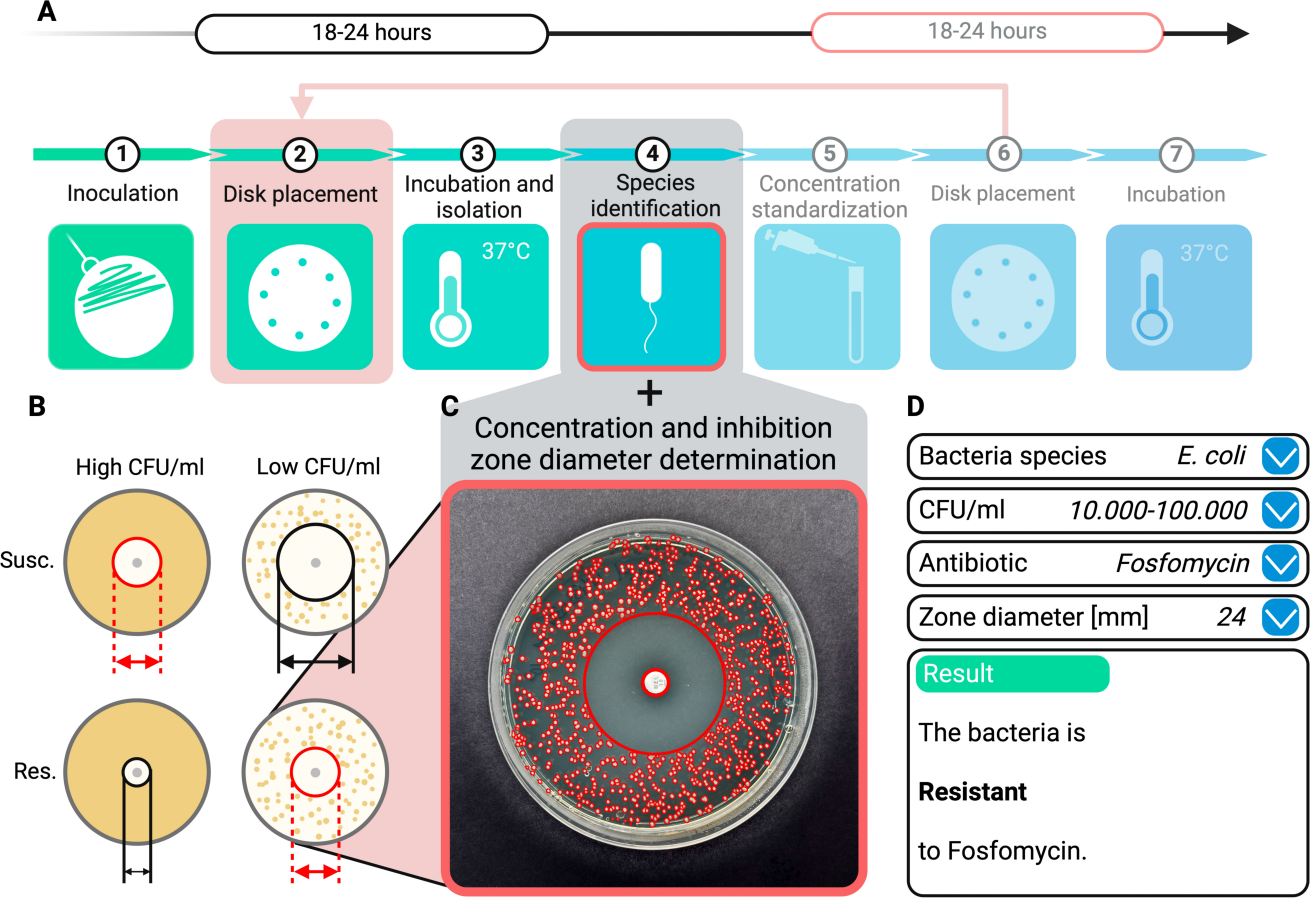
Comparison of conventional AST and concentration-adjusted disk diffusion DST method for urine diagnostics. (**A**) The conventional method requires two incubation periods, extending the process to 36–48 hours, while the new approaches apply antibiotic disks directly to urine on agar, reducing the time to 18–24 hours and eliminating the need for inoculum standardization. (**B**) Lower bacterial concentrations yield larger inhibition diameters, whereas higher concentrations produce smaller diameters, potentially causing overlap between susceptible (S) and resistant (R) populations without inoculum standardization. (**C**) The concentration-adjusted methods correlate halo size directly with bacterial concentration, removing the necessity for inoculum standardization. (**D**) An algorithm evaluates bacterial species, concentration, antibiotic type, and halo size to determine resistance behavior.

Direct susceptibility testing (DST) provides rapid phenotypic antimicrobial susceptibility results directly from patient samples, such as urine, without needing pathogen isolation, sub-culturing, and inoculum standardization, typically taking 18–24 hours and requiring fewer resources and skills (refer to Fig. 1A). DST significantly reduces turnaround time, allowing for faster initiation of targeted antimicrobial therapy, potentially leading to better treatment outcomes, improved patient care, and lower treatment costs (12–14). In addition, DST preserves the pathogen’s phenotypic characteristics by avoiding sub-culturing, which can alter the bacterial phenotype and lead to inaccurate results. DST also evaluates the antibiotic sensitivity of the entire sample, offering a comprehensive perspective on microbial resistance and enhancing the clinical relevance of the results (15, 16). Numerous studies have investigated DST across various sample types, generally finding a high level of agreement between standard AST and DST methods (17, 18). In particular, research focusing on urine samples comparing standard AST and DST using disk diffusion techniques has reported overall agreement rates typically exceeding 90% (19–24). However, despite these promising results, DST has certain limitations—one of which is its sensitivity to variations in bacterial concentration, which can compromise test accuracy if not accounted for.

It is well known that the size of the inhibition zone, or halo, and the MIC can be influenced by bacterial concentration. This phenomenon, known as the “inoculum effect,” is described as a significant decrease in halo size or an increase in the MIC when the inoculum size is increased (25–27). The inoculum effect varies between antibiotic and bacterial combinations (28–32). Without standardizing bacterial concentration, there is a risk of misclassification: resistant pathogens at low concentrations might be incorrectly identified as susceptible, while susceptible pathogens at high concentrations could be mistakenly deemed resistant (refer to Fig. 1B). Although there is no universally accepted threshold for colony-forming units per milliliter (CFU/mL) to define a UTI, some guidelines set the threshold at 10^3^ CFU/mL, which serves as the lower cutoff for a UTI in this study (33).

Despite the known influence of bacterial concentration on zone diameters, there is currently no established DST approach that systematically incorporates this factor into result interpretation. This gap limits the reliability of direct susceptibility testing, particularly at very high or low bacterial loads.

This research presents two adapted rapid and direct disk diffusion methods. Correlating bacterial concentration and halo size eliminates the need for standardized bacterial suspensions, streamlines the diagnostic process, and improves the efficiency of diagnosing bacteriuria and resistance patterns in patients with suspected UTIs.

## RESULTS

### Inoculum effect on inhibition zone diameter

**Fig 2:**
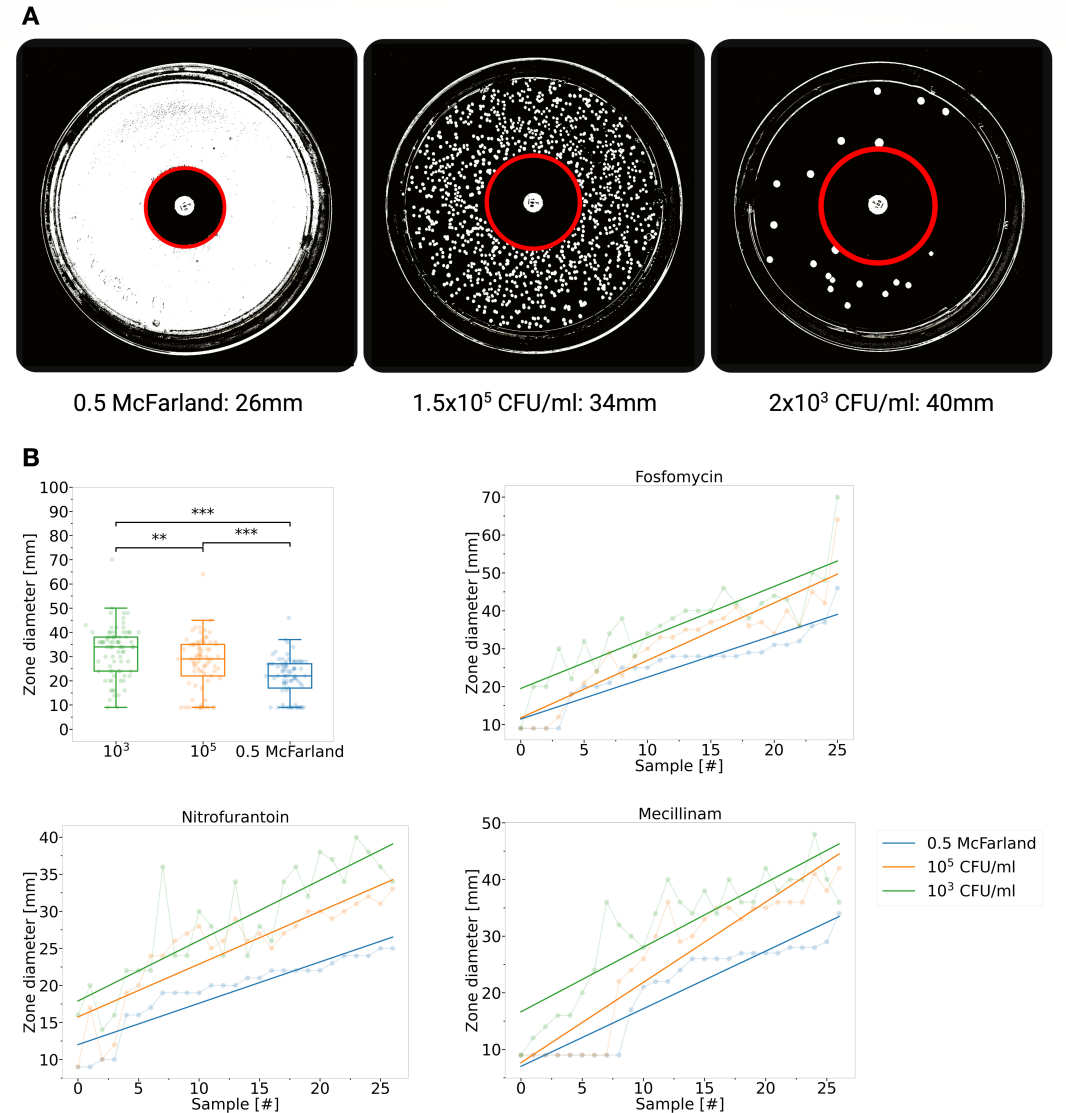
Correlation of inhibition zone diameter size with diluted bacterial suspensions. (**A**) The images illustrate an example of the relationship between bacterial concentration and halo size. (**B**) 0.5 McFarland suspensions of 27 clinical urine samples were diluted (10^5^ and 10^3^ CFU/mL) and assessed using fosfomycin, nitrofurantoin, and mecillinam. Notably, the inhibition halos for suspensions at 0.5 McFarland, 10^5^, and 10^3^ CFU/mL showed significant differences (*P* < 0.001 for 0.5 McFarland vs. 10^5^ and 0.5 McFarland vs. 10^3^ CFU/mL, *P* < 0.01 for 10^5^ vs. 10^3^ CFU/mL). Samples were further analyzed based on their inhibition halos, revealing a consistent pattern in halo sizes across different antibiotics and concentrations (fosfomycin: 0.5 McF vs. 10^5^ CFU/mL: r_S_ = 0.95, 0.5 McF vs. 10^3^ CFU/mL: r_S_ = 0.87, and 10^5^ vs. 10^3^ CFU/mL: r_S_ = 0.89, *P* < 0.001); nitrofurantoin: 0.5 McF vs. 10^5^ CFU/mL: r_S_ = 0.90, 0.5 McF vs. 10^3^ CFU/mL: r_S_ = 0.84, and 10^5^ vs. 10^3^ CFU/mL: r_S_ = 0.85, *P* < 0.001; mecillinam: 0.5 McF vs. 10^5^ CFU/mL: r_S_ = 0.94, 0.5 McF vs. 10^3^ CFU/mL: r_S_ = 0.81, and 10^5^ vs. 10^3^ CFU/mL: r_S_ = 0.85, *P* < 0.001).

To investigate the inoculum effect, inhibition zones of different bacteria isolated from clinical urine samples (*n* = 27) and suspended in phosphate-buffered saline (PBS) were measured on agar plates at various defined bacterial concentrations. Figure 2 illustrates that the inhibition zone diameters expanded as bacterial dilution increased. The analysis revealed that the distributions of inhibition zone diameters for all three suspensions and dilutions of clinical urine samples were non-normal. The non-parametric Kruskal-Wallis test identified significant differences in the inhibition zone diameters between the 0.5 McFarland suspensions and their dilutions, with *P* < 0.001, indicating high statistical significance. Furthermore, pairwise Mann-Whitney U comparisons supported these findings, showing *P*-values < 0.01.

The diameter of the inhibition zones for each antibiotic was analyzed in more detail. Spearman’s rank correlation coefficients were calculated, indicating a consistent trend where inhibition zone sizes of the suspensions and their dilutions increased synchronously.

These findings indicated a direct relationship, where diluting the bacterial concentration from 0.5 McFarland to 10^5^ to 10^3^ CFU/mL led to an increase in the average inhibition zone diameter across the tested levels.

**Fig 3:**
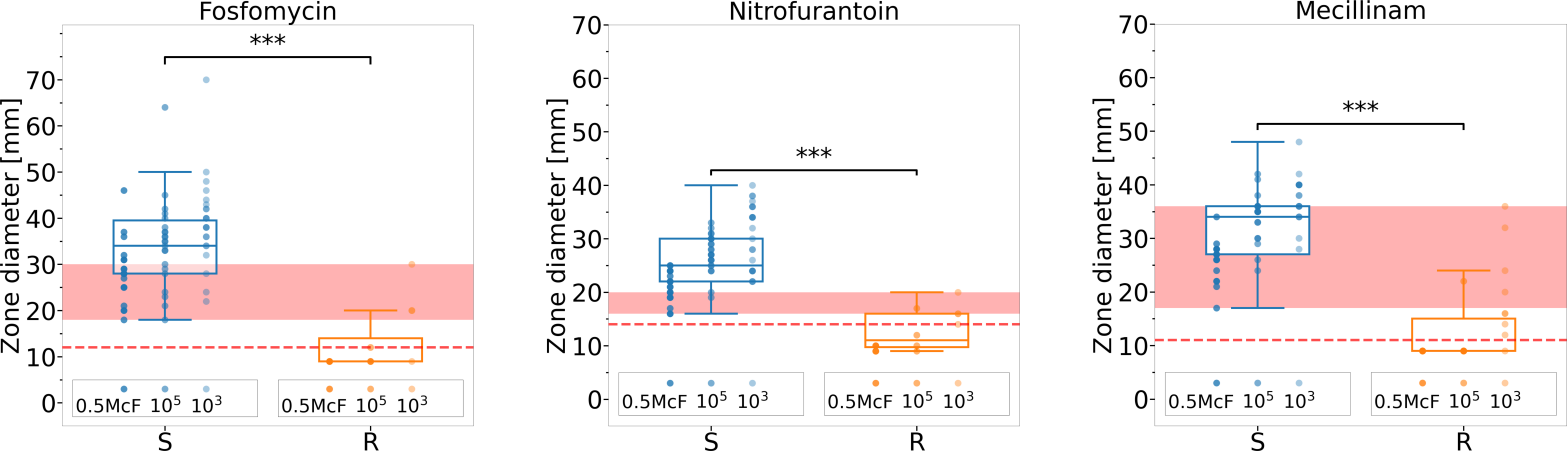
Inhibition zone diameters of susceptible and resistant urinary isolates at different bacterial concentrations. Suspensions of 27 urine isolates were tested at 0.5 McFarland, 10^5^, and 10^3^ CFU/mL against fosfomycin, nitrofurantoin, and mecillinam. Significant differences were observed between susceptible and resistant isolates (*P* < 0.001). However, overlapping inhibition zone diameters prevented a clear quantitative distinction between groups. The vertically arranged measuring points represent bacterial suspensions at concentrations of 0.5 McFarland, 10^5^ CFU/mL, and 10^3^ CFU/mL from left to right. The red line indicates the antibiotic-specific breakpoints defined by the CLSI or extrapolated from CLSI standards, while the red shaded area represents the overlap in halo sizes between susceptible and resistant bacteria tested. For tabulated data, see Table 1.

To further emphasize the importance of considering inoculum size and bacterial concentration or the need to standardize the inoculum, inhibition zone diameters were measured from PBS suspensions of bacteria isolated from 27 clinical urine samples. These suspensions were prepared at 0.5 McFarland, 10^5^, and 10^3^ CFU/mL concentrations and were tested against three antibiotics. The results were labeled as either susceptible or resistant, with resistance determined based on standard analysis (refer to Fig. 3; Table 1). This approach highlights the issue illustrated in Fig. 1B. Without incorporating the bacterial concentration of the bacterial suspension, there were overlaps between the halos of susceptible and resistant bacteria. This overlap complicated precise differentiation, especially in cases where susceptible bacteria were present in high concentrations and resistant bacteria were found in diluted suspensions. Based on CLSI-prescribed breakpoints or extrapolated thresholds derived from CLSI M100, 100% of the bacterial–antibiotic combinations tested were correctly identified as truly susceptible. However, only 64.7% of resistant cases were accurately classified. These misclassifications occurred across all three antibiotics tested. It is important to note that these figures reflect the entire set of analyzed suspensions (0.5 McFarland, 10^5^, and 10^3^ CFU/mL), although misclassifications were observed exclusively in the diluted suspensions.

**TABLE 1:** Differentiation of susceptible and resistant urinary isolates based on inhibition zone diameters alone^*a*^

Antibiotic	True susc. if ≥ [mm]	True susc. [%]	True resistant [%]
Fosfomycin	12	100.0	66.7
Nitrofurantoin	14	100.0	58.3
Mecillinam	11	100.0	66.7
**Total**		**100.0**	**64.7**

^*a*^ Bacterial suspensions (0.5 McFarland, 10⁵, and 10³ CFU/mL) from 27 urine samples were tested against fosfomycin, nitrofurantoin, and mecillinam. See Fig. 3 for graphical representation.

### Threshold-adapted approach for distinguishing resistance, incorporating bacterial concentration

**Fig 4:**
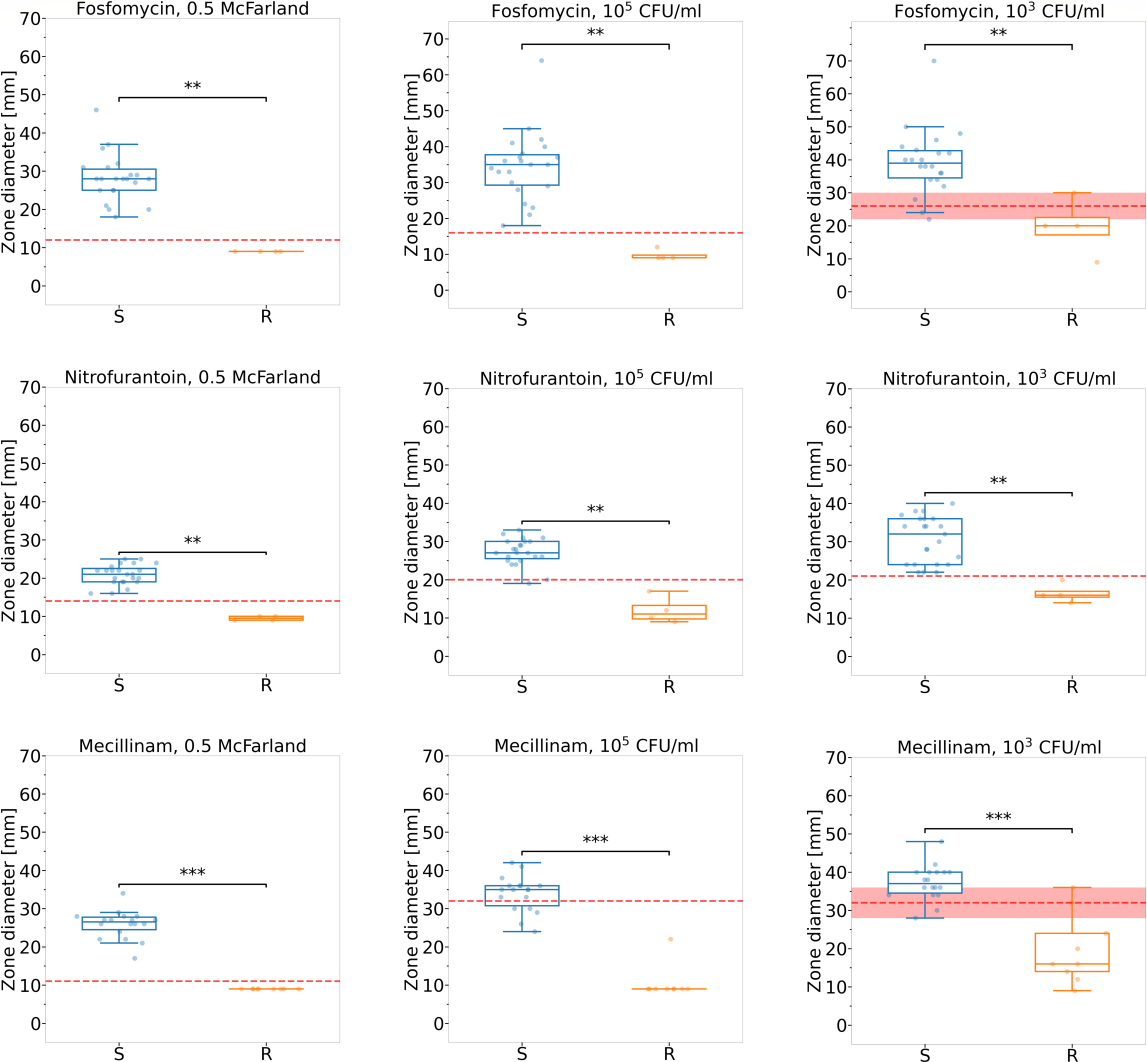
Inhibition zone diameters of urinary isolates stratified by antibiotic and bacterial concentration. Suspensions of 27 urine isolates were tested at 0.5 McFarland, 10^5^, and 10^3^ CFU/mL against fosfomycin, nitrofurantoin, and mecillinam. Thresholds for categorizing susceptibility were adapted for each antibiotic and concentration, specifically for the 10^5^ and 10^3^ CFU/ml suspensions. Significant differences were observed between susceptible and resistant isolates (*P* < 0.01 for fosfomycin and nitrofurantoin, *P* < 0.001 for mecillinam). Considering bacterial concentration improved quantitative differentiation. The red line shows the antibiotic-specific breakpoints defined by CLSI or extrapolated from CLSI standards for 0.5 McFarland or those that optimize differentiation between susceptible and resistant bacteria derived from reference strains (10^5^ and 10^3^ CFU/mL). The red shaded area represents the overlap in halo sizes between susceptible and resistant bacteria tested. For details, see numerical details, see Table 2.

The bacteria isolated from clinical urine samples were analyzed further. The boxplots in Fig. 4 illustrate the inhibition zone size, taking into account both the specific antibiotic tested and the bacterial concentration. By analyzing the relationship between inhibition zone size, antibiotic type, and bacterial concentration across eight reference strains, new threshold values were established to differentiate between susceptible and resistant bacteria at concentrations of 10^5^ and 10^3^ CFU/mL (see Fig. S1; Table S1). These revised thresholds were then applied to clinical urine isolates prepared in standardized suspensions of 10^5^ and 10^3^ CFU/mL. For samples adjusted to 0.5 McFarland, CLSI breakpoints or extrapolated criteria based on CLSI standards were used. Applying the new criteria yielded an overall categorical agreement of 94.7% in identifying susceptible bacteria, while resistant pathogens were correctly classified in 94.1% of cases. The specific categorical agreement rates for each antibiotic and bacterial concentration are presented in Table 2.

**TABLE 2:** Differentiation of susceptible and resistant urinary isolates by inhibition zone diameter and bacterial concentration^*a*^^,^^*b*^

Antibiotic	Susc. if ≥ [mm]			True susc. [%]			True resistant [%]		
	0.5MF	10^5^	10^3^	0.5MF	10^5^	10^3^	0.5MF	10^5^	10^3^
Fosfomycin	12	16	26	100.0	100.0	90.9	100.0	100.0	75.0
Nitrofurantoin	14	20	21	100.0	95.7	100.0	100.0	100.0	100.0
Mecillinam	11	32	32	100.0	72.2	88.9	100.0	100.0	77.8
**Total**				**94.7**			**94.1**		

^*a*^ Note: 0.5MF = 0.5 McFarland; 10^5^ = 10^5^ CFU/mL; 10^3^ = 10^3^ CFU/mL.

^*b*^ 27 urine isolates were tested against fosfomycin, nitrofurantoin, and mecillinam at three concentrations (0.5 McFarland, 10⁵, 10³ CFU/mL). Adapted thresholds for 10⁵ and 10³ suspensions were derived from reference strains; see Fig. 4 for graphical representation.

In addition to the clinical uropathogens, eight bacterial reference strains were tested at different concentrations and with various antibiotics. The boxplots, as well as the breakpoints for 0.5 McFarland bacterial suspensions, derived or extrapolated from the CLSI guidelines, along with the newly determined thresholds for the corresponding dilutions, can be found in Fig. S1 and Table S1. Except for one falsely resistant measurement point (fosfomycin at 10^3^ CFU/mL) and two falsely susceptible measurement points (mecillinam at 10^5^ CFU/mL and mecillinam at 10^3^ CFU/mL), the optimized thresholds were successful in accurately distinguishing between sensitive and resistant bacteria, resulting in 97.6% of the reference strain bacteria being correctly identified as susceptible and 96.3% as resistant.

A database was created using the breakpoints established from these reference strains. When clinical urine samples (*n* = 27) were applied to agar dishes and tested directly using the threshold-adapted DST approach, the resulting halos and concentrations were compared to the breakpoints in this reference database. This comparison revealed an agreement of 93.7% for true susceptibility and 94.1% for true resistance between the threshold-adapted DST and the standard disk diffusion method. Four samples were incorrectly categorized as resistant, and one sample, specifically *Enterococcus faecalis* with fosfomycin, was mistakenly classified as susceptible. Additionally, for one bacterium-antibiotic combination, *Staphylococcus aureus* with fosfomycin, no reference values were available, making it impossible to label it as resistant or susceptible (Fig. 6; threshold-adapted approach).

### Regression-based approach for predicting resistance profiles from inhibition zone sizes

In an alternative approach, a regression model was developed to correlate inhibition zone sizes from diluted bacterial suspensions with those from standard 0.5 McFarland suspensions. Linear regression was applied, using diluted halo size as the predictor and 0.5 McFarland halo size as the response. The model, developed with reference strains and tested with clinical urine samples, was evaluated by comparing predicted values with traditional disk diffusion measurements (refer to Fig. S2; Table S2).

In the initial approach, 27 uropathogens were isolated from clinical samples.

Standardized bacterial suspensions were prepared by adjusting cultures to a 0.5 McFarland turbidity standard. These suspensions were then serially diluted in PBS to achieve final concentrations of 10^5^ and 10^3^ CFU/mL. The prepared suspensions were tested using disk diffusion with three antibiotics: fosfomycin, nitrofurantoin, and mecillinam. Halo sizes from suspensions at concentrations of 10^5^ and 10^3^ CFU/mL were input into the regression model to calculate the corresponding inhibition zone diameters for 0.5 McFarland. For fosfomycin, both 10^5^ and 10^3^ CFU/mL concentrations achieved 100% accuracy in predicting susceptible bacteria correctly, with 75.0% categorical agreement for resistant bacteria at 10^3^ CFU/mL. Nitrofurantoin also showed 100% categorical agreement for both susceptible and resistant bacteria at both concentrations. For mecillinam, categorical agreement for susceptible isolates was 100% at both 10^5^ and 10^3^ CFU/mL, whereas categorical agreement for resistant isolates was 88.9% at 10^5^ and decreased to 55.6% at 10^3^ CFU/mL. Overall, the model correctly identified 100% of susceptible bacteria and 88.2% of resistant bacteria across all antibiotics.

**Fig 5:**
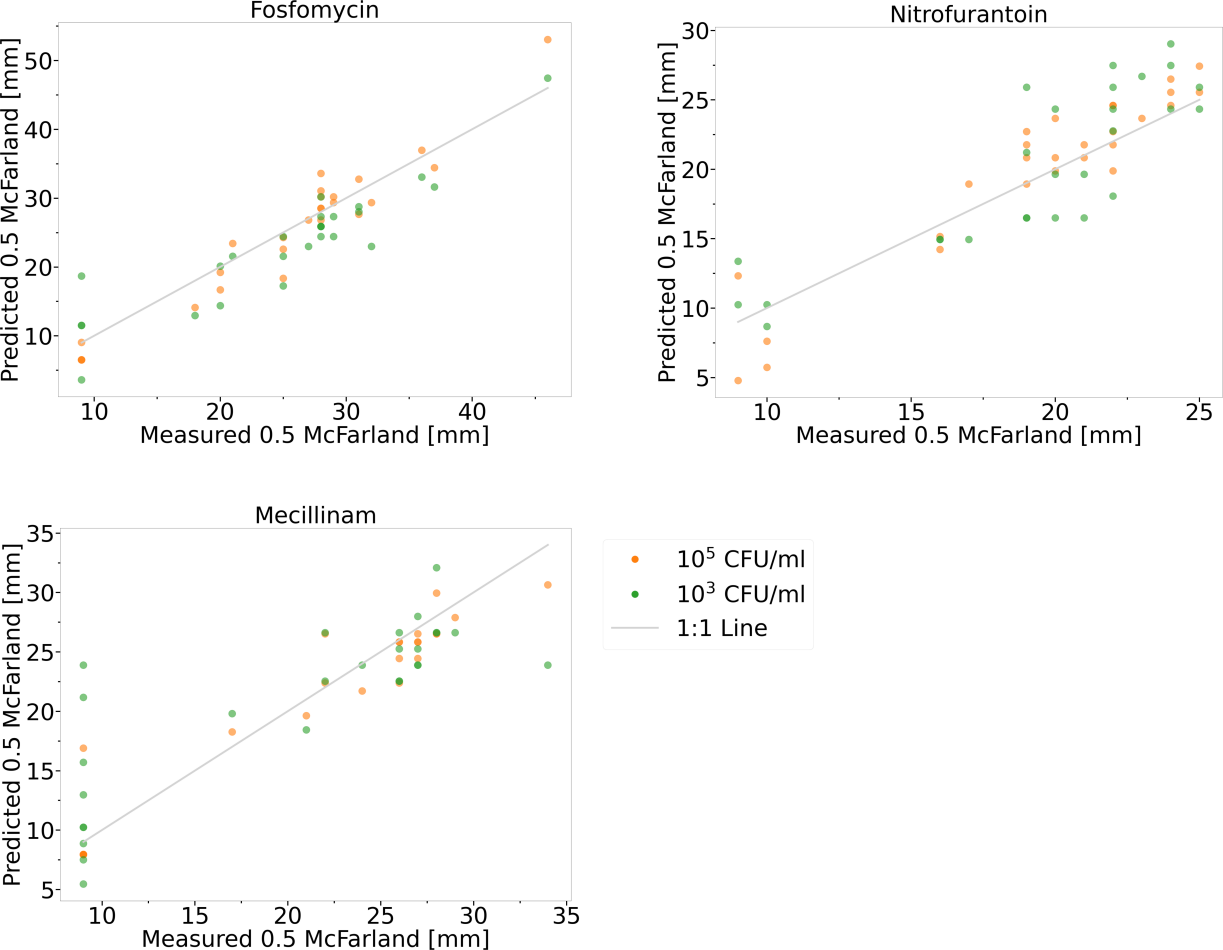
Comparison of measured and regression-based predicted inhibition zones for urinary isolates. Suspensions of 27 isolates at 10^5^ and 10^3^ CFU/mL were tested against fosfomycin, nitrofurantoin, and mecillinam. Regression models derived from reference strains were used to predict the corresponding inhibition zones at 0.5 McFarland, which were then plotted against the measured 0.5 McFarland values. The 1:1 line indicates the ideal scenario where predicted and measured halo sizes are identical. For statistical data, see Table 3.

The 10^5^ CFU/mL concentrations for all three antibiotics exhibited lower mean absolute error (MAE) values compared to the 10^3^ CFU/mL concentrations, indicating better prediction accuracy at higher concentrations (refer to Fig. 5; Table 3).

**TABLE 3:** Differentiation of susceptible and resistant urinary isolates based on regression models^*a*^^,^^*b*^

Antibiotic	MAE		True susc. [%]			True resistant [%]		
	10^5^	10^3^	0.5MF	10^5^	10^3^	0.5MF	10^5^	10^3^
Fosfomycin	2.34	3.47	100.0	100.0	100.0	100.0	100.0	75.0
Nitrofurantoin	1.82	2.59	100.0	100.0	100.0	100.0	100.0	100.0
Mecillinam	1.72	3.43	100.0	100.0	100.0	100.0	88.9	55.6
**Total**			**100.0**			**88.2**		

^*a*^ Note: MAE = mean absolute error; 0.5MF = 0.5 McFarland; 10⁵ = 10⁵ CFU/mL; 10³ = 10³ CFU/mL.

^*b*^ Zone diameters of 27 urine isolates at 10⁵ and 10³ CFU/mL were used to predict corresponding 0.5 McFarland values; see Fig. 5 for visualization.

**Fig 6:**
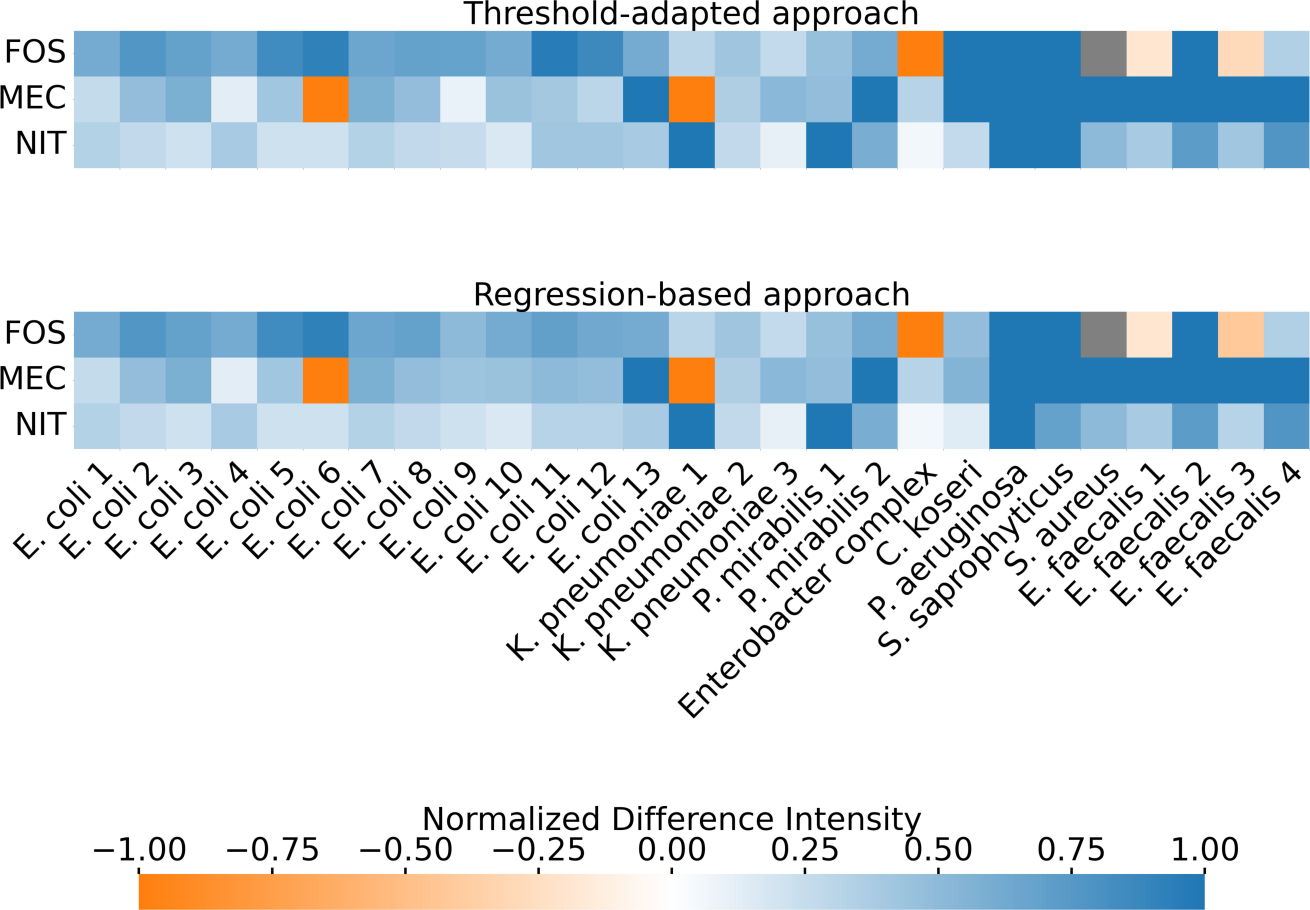
Normalized differences between measured or predicted inhibition zone diameters and breakpoint values. In the threshold-adapted approach, new breakpoints were defined for bacterial concentrations other than 0.5 McFarland. The halos from the DST were compared with the concentration-adjusted breakpoints. In the regression-based approach, the halos from the DST were measured, and the corresponding 0.5 McFarland halo sizes were calculated using regression models before being compared with the CLSI-defined breakpoints. The differences in the threshold-adapted method between the measured halo and concentration-adjusted breakpoint are slightly more pronounced than in the regression-based approach, which compares the predicted halo with the CLSI breakpoints. Still, overall, the same samples were correctly or incorrectly detected in both approaches. Bluish hues represent correct labeling of bacterial resistance or susceptibility, with deeper blue indicating a larger difference between the measured or calculated halo and breakpoint. Orange hues denote incorrect labeling, with deeper orange indicating a larger discrepancy. Normalization of these differences facilitates the comparison of resistance patterns across antibiotics and bacterial strains.

In a study performing DST on 27 clinical urine samples with varying bacterial concentrations, the analysis of the measured halos through regression models revealed that 93.7% of these samples were accurately classified as susceptible and 94.1% correctly as resistant. As with the threshold-adapted approach described in the previous section, four samples were misclassified as resistant, and one *E. faecalis* sample (labeled as E. faecalis 1) tested with fosfomycin was falsely identified as susceptible (Fig. 6; regression-based approach).

## DISCUSSION

The findings of this study demonstrate the potential of two adapted rapid direct disk diffusion methods for AST on bacteriuric urine samples, offering significant advantages in the context of diagnosing and managing suspected UTIs. These approaches eliminate the need for standardized bacterial suspensions by integrating bacterial concentration analysis directly into the AST process. The results underscore the methods’ effectiveness, with a high degree of categorical agreement observed in distinguishing between susceptible and resistant bacteria, although some limitations and areas for improvement were identified.

One of the most significant advantages of these methods is their ability to reduce the time to result. Traditionally, AST requires bacterial isolation and the preparation of standardized suspensions, which can delay the start of targeted antibiotic therapy. By integrating bacterial concentration analysis directly with the AST disk diffusion test, these methods streamline the diagnostic process, enabling quicker clinical decision-making. These approaches are also very cost-effective and resource-efficient, making them especially attractive for resource-limited settings where both rapid diagnostics and the optimal use of limited resources are crucial for effective patient care (34, 35).

In exploring the effectiveness of these methods, the study examined the role of bacterial concentration in influencing AST results. By preparing bacterial suspensions at defined concentrations, it was observed that the size of inhibition halos varied significantly depending on bacterial concentration, emphasizing the importance of accounting for this factor in disk diffusion susceptibility testing (25). This finding underscores the well-known inoculum effect, where varying bacterial loads can lead to different interpretations of susceptibility or resistance, potentially impacting clinical outcomes (29). Notably, when bacterial concentration was considered during the analysis of halo sizes in defined suspensions with bacteria isolated from clinical urine samples, the categorical agreement of identifying true resistant results improved significantly. The categorical agreement improved from 64.7% to 94.1% and 88.2% for the threshold-adapted and regression-based approaches, respectively, when compared to measuring halo size alone. This highlights the critical role of concentration in achieving accurate AST outcomes and reinforces the need for careful consideration of bacterial load in susceptibility testing.

Building on this understanding, the study further validated the described methods by directly applying clinical urine samples to agar plates, bypassing the need for standardized bacterial suspensions. Using the threshold-adapted method and a concentration-adjusted database of breakpoints derived from reference strain experiments, the results from this direct application method showed a 93.8% agreement with traditional AST protocols. This suggests that reliable results can be obtained from bacteriuric urine samples even without adjusting bacterial concentrations, provided there is robust reference data to guide the interpretation. The regression-based approach also demonstrated 93.8% agreement with the standard disk diffusion method when urine samples were applied directly to the agar plates and disk diffusion tests were performed without prior standardization of the inoculum. The results were less clear compared to the threshold-adapted approach. However, all urine samples were classified equally using both methods. Furthermore, while the threshold-adapted approach requires the establishment of new breakpoints, the regression-based method seems to integrate more easily with existing processes and standard operating procedures, as it does not require defining new breakpoints and can utilize standard reference breakpoint tables (10, 11, 36).

However, despite these promising results, the study also identifies several challenges that must be addressed. One of the primary issues is defining inhibition halos at low bacterial concentrations. At these lower concentrations, the edges of inhibition zones can become less distinct, complicating the interpretation of results (refer to Fig. 2A). This challenge is particularly pronounced with swarming pathogens like *Proteus mirabilis*, where the inhibition halos can be challenging to define due to the pathogen’s tendency to spread extensively across the medium. Such difficulties could lead to discrepancies between the rapid method and traditional AST.

Specifically, colonies were observed up to the antibiotic disk in samples labeled as falsely resistant, and the halo edges were poorly defined (refer to Fig. S3). This initial observation led to their classification as resistant. However, in the regular AST, these colonies often disappeared, and the halos became more sharply defined and fell above the breakpoints, resulting in their classification as sensitive. A potential explanation for this phenomenon lies in the nature of DST, which preserves the pathogen’s phenotypic characteristics by avoiding sub-culturing. Sub-culturing, required in traditional AST, can alter the bacterial phenotype, potentially leading to inaccurate results (15, 16). Specifically, sub-culturing can lead to lower MICs, meaning that *in vitro* MIC results may not accurately reflect the *in vivo* activity of the antibiotic (37, 38).

DST should be conducted exclusively on urine samples collected from patients who are not pre-treated with antibiotics. Pre-treatment with antibiotics can significantly alter the microbial composition and potentially inhibit bacterial growth, leading to inaccurate susceptibility results.

Integrating these insights into a unified diagnostic process highlights the methods’ potential for rapid, accurate AST, particularly in settings where time and resources are constrained. While considering bacterial concentration remains important for precise AST outcomes, the study demonstrates that the introduced methods can effectively streamline the diagnostic process, offering a practical solution that balances speed with accuracy.

Further refinement of the adapted breakpoints and the regression models and expanded validation across a broader range of pathogens and bacterial concentrations will be essential to maximize the method’s utility. Additionally, incorporating the concentration-adjusted DST approaches into automated systems could further enhance their applicability in various healthcare environments, ensuring that rapid and accurate diagnostics are more widely accessible.

## MATERIALS AND METHODS

### Reference strains and antibiotics

The bacterial reference strains used in this study were *Escherichia coli* ATCC 25922, *Klebsiella pneumoniae* ATCC 13883, *Proteus mirabilis* ATCC 14153, *Enterobacter hormaechei* ATCC 700323, *Pseudomonas aeruginosa* ATCC 27853, *Staphylococcus saprophyticus* ATCC 15305, *Staphylococcus aureus* ATCC 25923, and *Enterococcus faecalis* ATCC 29212, all obtained from AUROSAN GmbH. The antibiotic disks employed included fosfomycin 200 µg with glucose-6-phosphate 50 µg, acquired from Mast Group Ltd., and mecillinam 10 µg and nitrofurantoin 100 µg, sourced from Thermo Fisher Scientific. These antibiotics were selected following guidelines for treating uncomplicated UTIs (2, 33). Incubations were performed at 37°C for 18 hours.

### Reference database

Bacterial cultures were initiated by incubating the reference strains on LB agar (Sigma) for 18 hours. Following incubation, bacterial suspensions were prepared in phosphate-buffered saline (PBS, Sigma) to a concentration of 0.5 McFarland, with additional 10^3^ and 10^5^ dilutions in PBS. These suspensions were evenly spread onto Mueller-Hinton agar (Sigma) using a sterile cotton swab. To confirm that PBS did not influence antimicrobial activity measurements, we compared inhibition zone diameters obtained using PBS versus 0.9% saline suspensions. All eight reference strains included in this study were tested against the same three antibiotics (fosfomycin, nitrofurantoin, and mecillinam). PBS was chosen as the suspension medium because its ionic composition and buffering capacity more closely resemble the physiological properties of urine than saline, particularly in terms of pH stability. The mean absolute difference between PBS and saline was 0.88 mm, and the directional mean difference (PBS − saline) was −0.46 mm, indicating no systematic bias. Of the 24 comparisons, 23 (96%) showed differences of 2 mm or less. A single outlier (*E. hormaechei* with mecillinam) exhibited a 7 mm difference; however, this, and any other deviation, did not affect susceptibility categorization. An antibiotic disk was then placed at the center of each agar plate. After further incubation at 37°C for 18 hours, inhibition halos were measured to determine bacterial susceptibility. Only the inhibition zones from the 0.5 McFarland concentration were used to classify bacteria as sensitive or resistant based on the CLSI breakpoint tables. The dilutions were categorized based on the corresponding 0.5 McFarland suspension. The resulting data were then stored in a database, including details on the antibiotic tested, resistance classification, inhibition zone diameter, species, and bacterial concentration.

### Clinical urine samples

Urine samples were obtained from patients at the Klinikum rechts der Isar, Technical University of Munich, suspected of having a UTI. The Institute of Medical Microbiology, Immunology, and Hygiene conducted the initial microbiological analysis, including species identification through matrix-assisted laser desorption/ionization-time-of-flight (MALDI-TOF) analysis. Samples meeting the inclusion criteria—namely, a bacterial count of at least 10^3^ CFU/mL, mono-infections with bacteria from the *Enterobacterales*, *Staphylococcus*, *Enterococcus*, or *Pseudomonas* genera, and no prior antibiotic treatment—were selected for further study.

As dipstick or clinical symptom data were not available, the presence of 10^3^ CFU/mL was used to define bacteriuria. Therefore, the samples in this study are referred to as bacteriuric urine samples rather than confirmed UTIs.

Each sample was stored overnight at 4°C before processing. A 10 µL aliquot of each urine sample was applied to LB agar plates and evenly distributed using a sterile Drigalski spatula, following the spread plate technique. For initial urine plating and quantification of bacterial concentration, LB agar was used as a non-selective medium to enable accurate enumeration of CFU/mL. This approach ensured reliable quantification across a broad range of uropathogens. Plates were incubated for 18 hours at 37°C, and bacterial concentration was determined by manually counting visible colonies. Simultaneously, samples were evenly spread on Mueller-Hinton agar using a sterile cotton swab, and antibiotic disks were applied before incubation, representing the DST method.

Following the initial incubation, the LB agar plates were used to determine the concentration of bacteria in the urine, classifying them into categories of 1,000–10,000 CFU/mL, 10,000–100,000 CFU/mL, or *>*100,000 CFU/mL. These plates were then used to prepare standardized 0.5 McFarland suspensions and their respective 10^3^ and 10^5^ dilutions in PBS. Since a 0.5 McFarland standard corresponds approximately to a bacterial suspension of 1–2 × 10^8^ CFU/mL, these dilutions are referred to as 10^5^ and 10^3^ CFU/mL suspensions in this study (39). These suspensions were then subjected to further incubation on Mueller-Hinton agar, with antibiotic disks applied to the plates. The inhibition zones on the Mueller-Hinton plates from the previous day were measured directly.

Results from the 0.5 McFarland suspensions, obtained the next day, were used as the standard disk diffusion test results for comparison with the direct inoculation method. Based on these measurements, bacterial isolates were classified as resistant or susceptible. Furthermore, the halo sizes of the 0.5 McFarland suspensions were arranged in ascending order and plotted alongside their respective dilutions to explore the relationship between bacterial concentration and halo size.

Each clinical isolate and reference strain was tested once per condition. Although technical replicates were not performed due to resource constraints, all experiments followed standardized procedures to ensure consistency and reproducibility.

The clinical urine samples were handled in accordance with the Declaration of Helsinki and received approval from the Ethics Committee of the Technical University Hospital of Munich (427/21 S-KH).

### Halo measurement and resistance classification

The halo measurements for the dilutions of the 0.5 McFarland suspensions followed specific guidelines. For the first dilution series, with an average concentration of approximately 10^5^ CFU/mL, the diameters of two opposite colonies were measured to determine the halo size. For the second dilution series, with an average concentration of approximately 10^3^ CFU/mL, the distance from the disk’s center to the nearest colony edge was measured and then doubled to determine the halo diameter. Inhibition zone diameters and distances were assessed visually with the naked eye. Measurements were taken from the bottom of the petri dish after incubation, using a caliper, while holding the plate against a dark background. Considering colonies within the halo was particularly relevant for the 0.5 McFarland suspensions. When evaluating colonies within the halo, we followed the recommendations provided by CLSI, measuring the distance from the colonies to the antibiotic disk. Exceptions were made if the halo was clearly defined and if colonies appeared only sporadically, as the clinical significance of these colonies is questionable, particularly when testing *E. coli* with fosfomycin (40). The breakpoints were established following CLSI tables due to their broad range of breakpoint values. To simplify the process, intermediate values were classified as resistant (10). This approach simplified the evaluation of categorical agreement and, given the limited sample size and exploratory nature of the study, allowed for clearer comparisons without introducing statistical sparsity in the intermediate category. However, it may slightly overestimate resistance rates.

For certain bacterium–antibiotic combinations, no breakpoints were available in the CLSI M100. In these cases, results were either interpreted by extrapolating breakpoints or, when no interpretation was feasible, they were considered based on the presence or absence of an inhibition zone; if no inhibition zone at all was observed, indicating a lack of detectable antimicrobial activity, thus resistance of the respective sample. For *S. aureus* ATCC 25923 tested with fosfomycin, the zone diameter breakpoints defined by Lu et al. (2011) were applied (41). In the case of fosfomycin and mecillinam, we interpreted results using extrapolated breakpoints, although we acknowledge that CLSI breakpoints for both agents are validated only for *E. coli*, with fosfomycin further restricted to urinary tract isolates. Importantly, extrapolation was applied only to other species within the Enterobacterales order. While EUCAST extends fosfomycin and mecillinam breakpoints to include species such as *Citrobacter* spp., *Klebsiella* spp., *Enterobacter* spp., and *P. mirabilis*, our analyses are based on CLSI standards, and any extrapolated interpretations are transparently reported.

The term “categorical agreement,” used to quantify the performance of the developed DST methods, refers to the percentage of samples that are correctly classified as resistant (including intermediate values as defined by CLSI) or susceptible, based on comparison with the reference results obtained through standard AST.

### Threshold optimization and data analysis

An algorithm was developed to optimize the thresholds for distinguishing between susceptible and resistant bacteria, specifically for suspensions of 10^5^ and 10^3^ CFU/mL. This optimization was based on eight reference strains. The algorithm aimed to correctly classify as many samples as possible (Fig. S1; Table S1). Concentration-dependent breakpoints for various antibiotics relevant to the *Enterobacterales*, *Staphylococcus*, *Enterococcus*, and *Pseudomonas* reference genera were compiled into a database and validated with clinical urine samples. A user-friendly graphical interface was developed using Python 3.9.7 to provide access to the database (refer to Fig. 1D).

Inhibition zones from clinical isolates prepared in defined 10^5^ and 10^3^ suspensions, as well as from directly inoculated clinical urine samples, were compared against the reference strain database and the standard disk diffusion test results. Additionally, the inhibition zones from defined PBS-suspended clinical isolates were visualized using boxplots in a two-step process. Initially, the measured inhibition zone diameter was the only factor considered to differ between susceptible and resistant bacteria. In a subsequent step, both the halo and the bacterial concentration in the suspension were considered.

### Predictive model development

To relate the diluted halo measurements to the standard 0.5 McFarland measurements, a regression-based modeling approach was employed. For each antibiotic, the paired data points were formed by taking the diluted halo size as the predictor variable (x) and the 0.5 McFarland halo size as the response variable (y). Linear regression models were first fitted to the data to estimate parameters of the form Halo_0.5McF_ = *a* + *b* × Halo_dilution_.

These models were developed using eight reference strains and subsequently tested with clinical urine samples from patients diagnosed with UTIs (refer to Fig. S2). This allowed for an assessment of its predictive power in real-world clinical settings. The model’s performance was assessed by comparing the predicted 0.5 McFarland values to the traditional disk diffusion protocol measurements from the clinical samples. This assessment included direct measurements of clinical urine samples, as well as testing defined suspensions and dilutions spiked with clinical isolates.

Categorical agreement metrics, including overall classification accuracy and antibiotic-specific categorical agreement for susceptible and resistant bacterial classifications based on CLSI predefined thresholds or extrapolated breakpoints derived from CLSI standards, were calculated to evaluate the model’s effectiveness in predicting resistance profiles. Here, as well, a graphical user interface was developed to facilitate the application of the model (refer to Fig. 1D).

### Statistics and reproducibility

The Shapiro-Wilk test was conducted to evaluate the normality of the data. The non-parametric Kruskal-Wallis test was utilized to assess the relationship between the diameter of the inhibition zone and the concentration of bacterial suspension. The Spearman rank correlation coefficient was computed to investigate this correlation further. The Mann-Whitney U test was used to compare differences between susceptible and resistant urine samples and between bacterial suspensions with varying bacterial loads. Statistical significance was denoted by asterisks, with the following thresholds: *P < 0.05; **P < 0.01; ***P < 0.001; ns = not significant.

Linear regression models were employed to predict inhibition zone diameters in 0.5 McFarland bacterial suspensions and assess bacterial resistance based on halo size measurements at different bacterial concentrations. The goodness of fit for each regression model was assessed using the R-squared statistic. The MAE measures the average absolute difference between the predicted and actual halo sizes, quantifying the accuracy of the model’s predictions.

The heatmap visualizes the accuracy of DST by normalizing halo diameters against predefined breakpoints. Differences between measured halos and breakpoints were scaled using maximum and minimum halo diameters for values above and below the breakpoint, respectively. The normalized values were mapped using a custom colormap from blue to orange, where bluish hues indicate correct labeling with more distinct patterns, and orange hues indicate incorrect labeling with more significant discrepancies.

## Data Availability

The data from the study, which encompass details about urine samples, halo dimensions, resistance categories, and bacterial concentrations, are saved in distinct CSV files. Access to the images of the agar dishes analyzed in this study can be granted upon request, particularly for the purposes of review of findings. For enhanced visual clarity in this publication, selected images (Fig. 1 and 2; Fig. S3) have been partially edited using GIMP 2.10.14 software. These edits were applied to improve visual representation without altering the scientific integrity or meaning of the data.

## SUPPLEMENTAL MATERIAL

Data S1 spectrum.00888-25-s0001.csv Standard disk diffusion AST results of reference strains.

Data S2 spectrum.00888-25-s0002.csv Direct Susceptibility Testing of clinical samples.

Data S3 spectrum.00888-25-s0003.csv Standard disk diffusion AST results of clinical samples.

Data S4 spectrum.00888-25-s0004.csv Sample IDs and concentrations.

Data S5 spectrum.00888-25-s0005.csv Inhibition zone diameters (in mm) for reference bacterial strains tested against three antibiotics, comparing measurements obtained using PBS and 0.9% saline as suspension media.

Fig. S1 spectrum.00888-25-s0006.tiff Differentiation between susceptible and resistant bacteria in reference strains, based on inhibition zone diameter, antibiotic tested, and bacterial concentration.

Fig. S2 spectrum.00888-25-s0007.tiff Linear regression models based on reference strains and R2-values.

Fig. S3 spectrum.00888-25-s0008.tiff Comparison of direct and standard susceptibility testing.

Supplemental material spectrum.00888-25-s0009.pdf Legends for supplemental figures and tables.

Supplemental tables spectrum.00888-25-s0010.docx Tables S1 and S2.

ASM does not own the copyrights to Supplemental Material that may be linked to, or accessed through, an article. The authors have granted ASM a non-exclusive, world-wide license to publish the Supplemental Material files. Please contact the corresponding author directly for reuse.
