# Supplementary figures and images for: Rapid direct disk diffusion testing for antibiotic resistance in urinary tract infections: a bacterial concentration-adjusted approach
Source: Microbiol Spectr. 2025 Sep 22;13(11):e00888-25. doi: 10.1128/spectrum.00888-25 (PMC12584718; doi:10.1128/spectrum.00888-25)

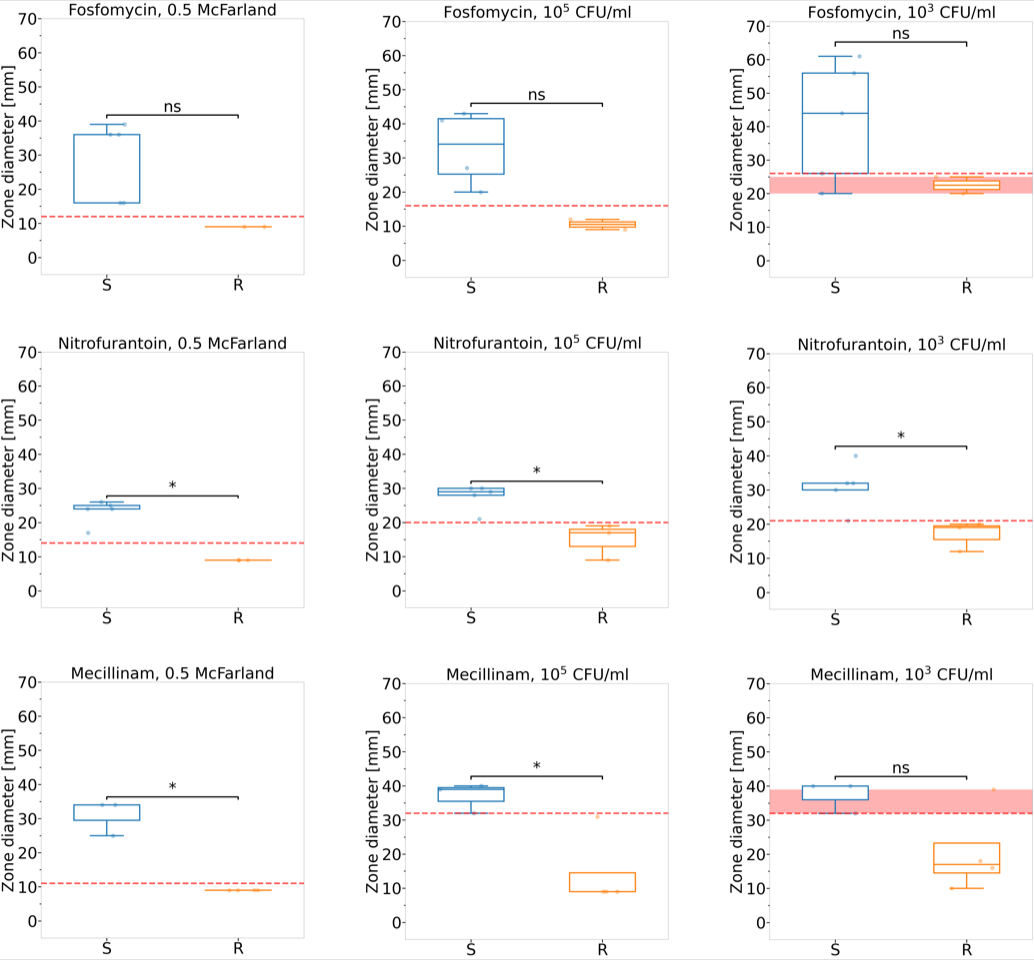

Supplement: Fig. S1 — Differentiation between susceptible and resistant bacteria in reference strains, based on inhibition zone diameter, antibiotic tested, and bacterial concentration. [file spectrum.00888-25-s0006.tiff]

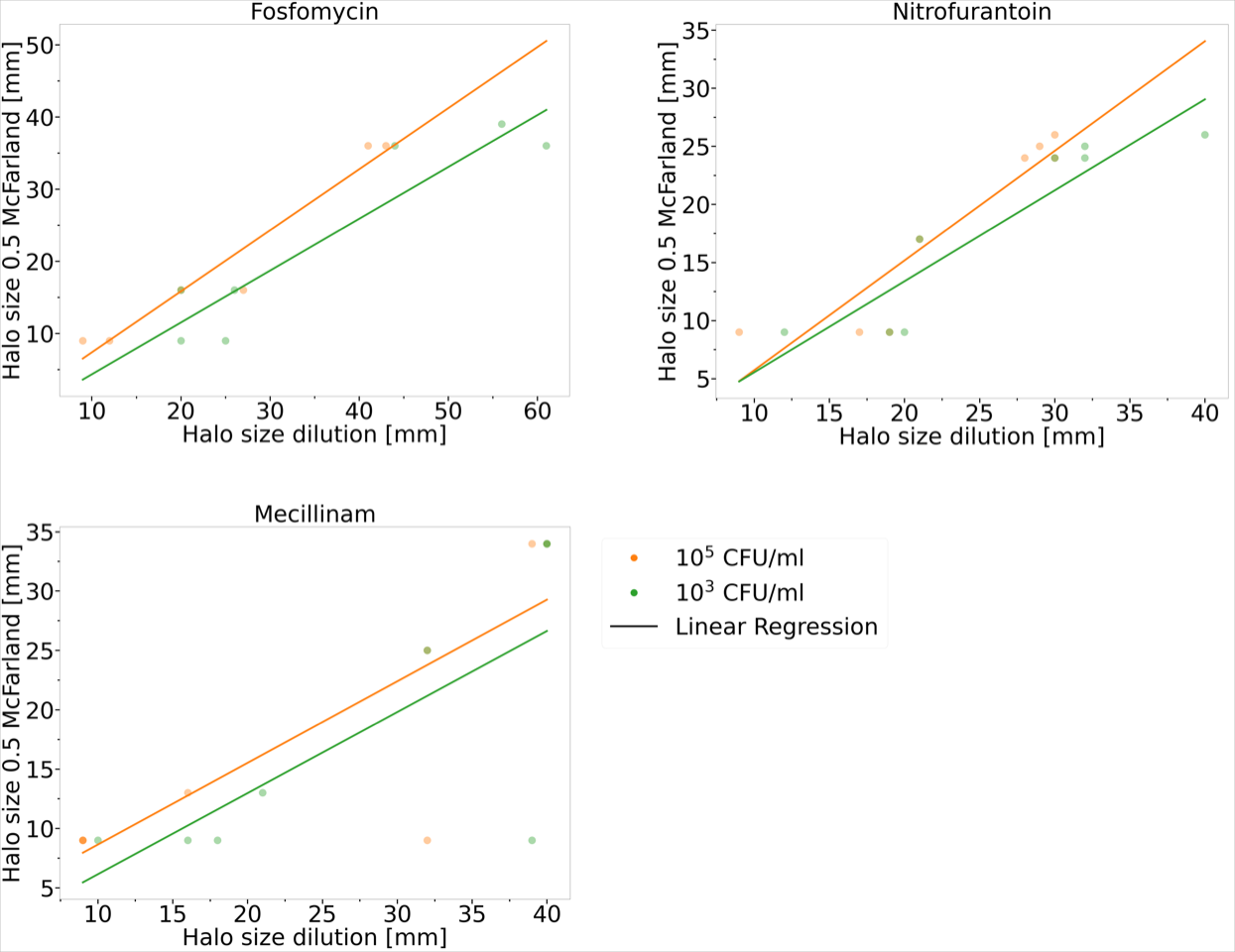

Supplement: Fig. S2 — Linear regression models based on reference strains and R2-values. [file spectrum.00888-25-s0007.tiff]

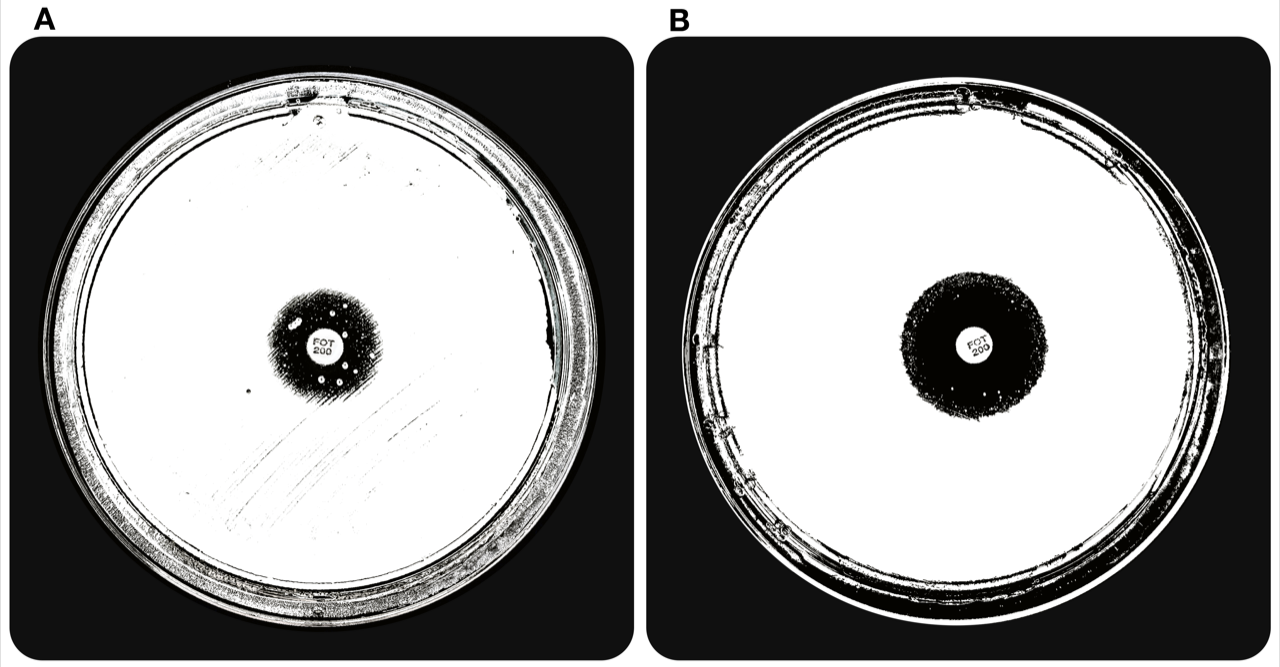

Supplement: Fig. S3 — Comparison of direct and standard susceptibility testing. [file spectrum.00888-25-s0008.tiff]
