# Supplementary material for: Rapid direct disk diffusion testing for antibiotic resistance in urinary tract infections: a bacterial concentration-adjusted approach
Source: Microbiol Spectr. 2025 Sep 22;13(11):e00888-25. doi: 10.1128/spectrum.00888-25 (PMC12584718; doi:10.1128/spectrum.00888-25)
Supplement: Supplemental material — Legends for supplemental figures and tables. [file spectrum.00888-25-s0009.pdf]

Supplemental figure and table captions

Figure S1 and Table S1

Differentiation between susceptible and resistant bacteria in reference strains, based on inhibition zone diameter, antibiotic tested, and bacterial concentration. Bacterial suspensions of eight reference strains at concentrations of 0.5 McFarland, 10^5, and 10^3 CFU/ml were subjected to tests against fosfomycin, nitrofurantoin, and mecillinam. Thresholds for distinguishing between susceptible and resistant bacteria were adapted for each antibiotic, specifically for the 10^5 and 10^3 CFU/ml suspensions. Analysis showed significant differences in inhibition zones between susceptible and resistant bacterial samples for only certain antibiotic-bacterial concentration combinations ($p < 0.05$ for nitrofurantoin and mecillinam at 0.5 McFarland and 10^5 CFU/ml). However, the results were not statistically significant for fosfomycin and mecillinam at a concentration of 10^3 CFU/ml. The red line shows the antibiotic-specific breakpoints defined by CLSI for 0.5 McFarland or those that optimize differentiation between susceptible and resistant bacteria (10^5 and 10^3 CFU/ml). The red shaded area represents the overlap in halo sizes between susceptible and resistant bacteria tested.

Figure S2 and Table S2

Linear regression models based on reference strains and R^2-values. The 10^5 and 10^3 CFU/ml bacterial dilutions of eight reference strains were plotted against their corresponding 0.5 McFarland suspensions for each antibiotic tested. Regression models were derived from these plots to predict the 0.5 McFarland halo sizes of clinical urine samples.

Figure S3

Comparison of direct and standard susceptibility testing. A When applying urine and antibiotic disk directly to the agar plate, the inhibition halo appeared less defined, with single colonies growing within the halo up to the disk. **B** When isolating bacteria from the urine and preparing a 0.5 McFarland suspension, the inhibition halo appeared more distinct, with only very small colonies within the halo, typically above the CLSI or extrapolated susceptibility breakpoint.
