# Supplementary material for: Rapid direct disk diffusion testing for antibiotic resistance in urinary tract infections: a bacterial concentration-adjusted approach
Source: Microbiol Spectr. 2025 Sep 22;13(11):e00888-25. doi: 10.1128/spectrum.00888-25 (PMC12584718; doi:10.1128/spectrum.00888-25)
Supplement: Supplemental tables — Tables S1 and S2. [file spectrum.00888-25-s0010.docx]

|  | Susc. if >=[mm] | | | True susc. [%] | | | True resistant [%] | | |
| --- | --- | --- | --- | --- | --- | --- | --- | --- | --- |
| Antibiotic | 0.5MF | 10^5^ | 10^3^ | 0.5MF | 10^5^ | 10^3^ | 0.5MF | 10^5^ | 10^3^ |
| Fosfomycin | 12 | 16 | 26 | 100.0 | 100.0 | 80.0 | 100.0 | 100.0 | 100.0 |
| Nitrofurantoin | 14 | 20 | 21 | 100.0 | 100.0 | 100.0 | 100.0 | 100.0 | 100.0 |
| Mecillinam | 11 | 32 | 32 | 100.0 | 100.0 | 100.0 | 100.0 | 100.0 | 75.0 |
| *Total* |  |  |  | *97.6* | | | *96.3* | | |
| Note: 0.5MF = 0.5 McFarland; 10^5^ = 10^5^ CFU/ml; 10^3^ = 10^3^ CFU/ml | | | | | | | | | |
|  |  |  |  |  |  |  |  |  |  |
| **Table S1:** For caption see section 'Figure and table captions' *Figure S1 and Table S1* | | | | | | | | | |

|  | R^2 | | Regression model | |
| --- | --- | --- | --- | --- |
| Antibiotic | 10^5^ | 10^3^ | 10^5^ | 10^3^ |
| Fosfomycin | 0.94 | 0.87 | -1.1019 + 0.8461x | -2.8639 + 0.7184x |
| Nitrofurantoin | 0.85 | 0.84 | -3.7195 + 0.9440x | -2.2947 + 0.7833x |
| Mecillinam | 0.70 | 0.53 | 1.7683 + 0.6874x | -0.6852 + 0.6828x |
| *Note*: 10^5^ = 10^5^ CFU/ml; 10^3^ = 10^3^ CFU/ml; *x* = inhibition zone diameter [mm] | | | | |
|  |  |  |  |  |
| **Table S2:** For caption see section 'Figure and table captions' *Figure S2 and Table S2* | | | | |
